# Intestinal current measurement detects age-dependent differences in CFTR function in rectal epithelium

**DOI:** 10.3389/fphar.2025.1537095

**Published:** 2025-02-24

**Authors:** Simon Y. Graeber, Olaf Sommerburg, Yin Yu, Julian Berges, Stephanie Hirtz, Heike Scheuermann, Jasmin Berger, Julia Duerr, Marcus A. Mall

**Affiliations:** ^1^ Department of Pediatric Respiratory Medicine, Immunology and Critical Care Medicine, Charité - Universitätsmedizin Berlin, Corporate Member of Freie Universität Berlin and Humboldt-Universität, Berlin, Germany; ^2^ German Center for Lung Research (DZL), Associated Partner Site Berlin, Berlin, Germany; ^3^ German Center for Child and Adolescent Health (DZKJ), Partner Site Berlin, Berlin, Germany; ^4^ Division of Pediatric Pulmonology, Allergy and Cystic Fibrosis Center, Department of Pediatrics, University Hospital Heidelberg, Heidelberg, Germany; ^5^ Department of Translational Pulmonology, Translational Lung Research Center, Member of the German Center for Lung Research, University of Heidelberg, Heidelberg, Germany

**Keywords:** CFTR, intestinal current measurement, rectal epithelium, age-dependency, CFTR modulator therapy, secretory diarrhea

## Abstract

**Objective:**

Intestinal current measurement (ICM) provides a sensitive bioassay for assessment of cystic fibrosis transmembrane conductance regulator (CFTR) function in rectal biopsies *ex vivo* and is used as a diagnostic tool for cystic fibrosis (CF). Furthermore, ICM was shown to be sensitive to detect pharmacological rescue of CFTR function by CFTR modulators in people with CF carrying responsive *CFTR* mutations. Results from clinical trials of CFTR modulators across age groups indicate that CFTR function in the sweat duct may be age-dependent with children reaching higher levels than adults. However, little is known about age dependency of CFTR function in the intestinal epithelium.

**Methods:**

We investigated CFTR-mediated chloride secretion in rectal biopsies from 258 people without CF and 72 people with pancreatic-insufficient CF from 1 month to 68 years of age. Change in transepithelial short-circuit current in response to cyclic adenosine monophosphate (cAMP)-mediated (100 μM IBMX, 1 µM forskolin, basolateral) and cholinergic (100 μM carbachol, basolateral) stimulation was assessed as a readout for CFTR function using perfused micro-Ussing chambers. Furthermore, quantitative real-time PCR of *CFTR* and morphometric analysis of epithelial cells lining the crypts and surface of the rectal mucosa were performed to assess regulation at the levels of gene expression and epithelial cell densities.

**Results:**

We found that CFTR-mediated chloride secretion across rectal tissues, as determined from cAMP-mediated as well as cholinergic chloride-secretory responses was highest during infancy and early childhood and declined with age in people without CF (both P < 0.001). Although, there was no difference in cAMP-mediated currents in people with CF, potassium-secretory responses induced by cholinergic stimulation were also reduced with increasing age. Transcript analyses showed that *CFTR* mRNA expression was slightly increased with increasing age in people without CF (P < 0.05). Morphometric analyses demonstrated that CFTR expressing colonocytes at the crypt base were decreased with age (P < 0.05). A secondary analysis of the ICM data of our previous studies on the effects of lumacaftor/ivacaftor on CFTR function in *F508del* -homozygous people with CF aged 12 years and older and 2–11 year old children showed correlations of the change in cAMP-mediated and cholinergic chloride secretory response with the age of people with CF (P < 0.01 and P < 0.05, respectively).

**Conclusion:**

These results demonstrate that CFTR function in the rectal epithelium is reduced with increasing age and indicate that this change is likely due to a decline in the number of secretory colonocytes at the crypt base. These findings suggest that differences in CFTR expressing cells may explain increased functional responses to CFTR modulator therapies in children compared to adult people with CF.

## 1 Introduction

**FIGURE 1:**
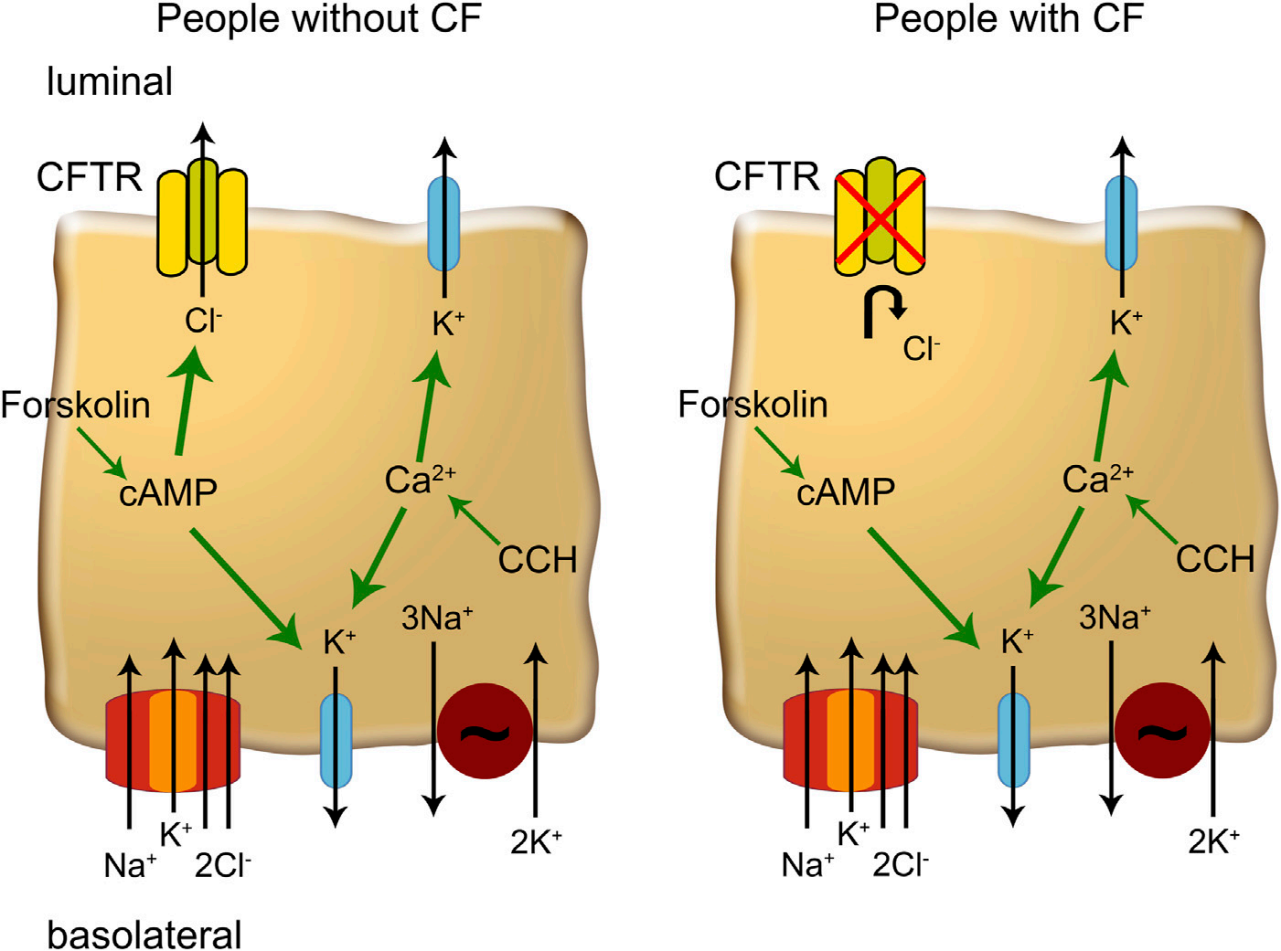
The mechanisms of ion transport in colonic enterocytes. Forskolin increases cytosolic cAMP which enhances CFTR (cystic fibrosis transmembrane conductance regulator)-conductance and increases basolateral K^+^ conductance. Carbachol (CCH) increases cytosolic Ca^2+^ concentration that activates K^+^ channels in the luminal and basolateral membrane, increasing the driving force for chloride secretion via CFTR. In people with CF, CFTR channels cannot be activated and Ca^2+^ mobilizing agonists enhance K^+^ secretion.

Cystic fibrosis (CF) is a hereditary disorder caused by mutations in the *CFTR* (cystic fibrosis transmembrane conductance regulator) gene, which encodes a chloride and bicarbonate channel crucial for maintaining the balance of ion and water transport across epithelial surfaces (Mall et al., 2024; Saint-Criq and Gray, 2017). Key target organs of CF are the lungs, the pancreas and the intestine (Grasemann and Ratjen, 2023). In the airways, CFTR dysfunction leads to impaired anion (chloride and bicarbonate) secretion and enhanced sodium absorption through the epithelial sodium channel (ENaC), resulting in hyperconcentrated and highly visco-elastic mucus (Boucher, 2019; Mall et al., 1998a). This abnormal mucus causes chronic airway infection and inflammation leading to progressive structural lung damage (Boucher, 2007). In the pancreas, CFTR is important for chloride and bicarbonate secretion in the pancreatic ducts. CFTR dysfunction causes hyperconcentration of pancreatic secretions and plugging of the ducts, leading to a backlog of digestive enzymes and auto-digestion of pancreatic tissue, which in turn causes severe pancreatitis and fibrosis with exocrine pancreatic insufficiency already present in ∼85% of infants with CF (Wilschanski and Novak, 2013; Ramsey and Galante, 2024). In the intestine, CFTR plays a key role in the regulation of cAMP-regulated chloride and fluid secretion essential for hydration of the mucus layer and lubrication of the intestinal surface (Greger et al., 1997; Greger, 2000; Kunzelmann and Mall, 2002; Mall et al., 1999). CFTR mediated chloride secretion in the intestine can be stimulated by forskolin via an increase in intracellular cAMP concentration (Figure 1) (Greger et al., 1997; Greger, 2000; Kunzelmann and Mall, 2002). Chloride secretion can be further increased by carbachol, a cholinergic agonist that activates calcium-regulated potassium channels increasing the driving force for apical chloride secretion (Greger et al., 1997; Greger, 2000; Kunzelmann and Mall, 2002). In CF, impaired chloride secretion leads to dehydration/hyperconcentration of intestinal mucus which can lead to severe bowel obstruction that can manifest as meconium ileus after birth or severe constipation leading to distal intestinal obstruction syndrome (DIOS) in older patients (Greger et al., 1997; Greger, 2000; Kunzelmann and Mall, 2002; Mall et al., 1999; Abraham and Taylor, 2017; Geibel, 2005). Beyond CF, as a key regulator of intestinal fluid homeostasis, CFTR, is also implicated in other intestinal disorders including secretory diarrhea, chronic constipation and colorectal cancer (CRC) (Thiagarajah et al., 2015; Chang et al., 2023; Spelier et al., 2024; Shi et al., 2021).

The development of CFTR modulators in recent years presents the first therapy to treat the basic defect of CF (Graeber and Mall, 2023). CFTR modulators aim to correct defective CFTR protein, either by improving its folding and trafficking to the cell membrane (e.g., elexacaftor, lumacaftor, tezacaftor) or by enhancing its gating function (e.g., ivacaftor) (Mall et al., 2024). The triple combination therapy elexacaftor/tezacaftor/ivacaftor (ETI) has shown remarkable clinical efficacy in people with CF and at least one *F508del*-*CFTR* allele as well as a range of other *CFTR* mutations (Middleton et al., 2019; Heijerman et al., 2019; Burgel et al., 2024). Real world observational studies showed that ETI therapy improves CFTR function to 40%–50% of normal CFTR activity in the intestinal epithelium and leads to a substantial improvement in lung function, lung ventilation, mucus plugging in the airways as well as airway dysbiosis and inflammation (Nichols et al., 2023; Graeber et al., 2022a; Graeber et al., 2022b; Schaupp et al., 2023; Stahl et al., 2024a). Interestingly, sweat chloride concentration measurements as a biomarker of CFTR function suggests an age dependent effect of ETI. In studies in 2–5 year old children homozygous for *F508del*, approximately 60% of children achieved sweat chloride levels below 30 mmol/L (Goralski et al., 2023), whereas the mean sweat chloride concentration in adolescent and adult people with CF was 48.0 mmol/L after ETI therapy (Heijerman et al., 2019). Similarly, the CFTR dual combination lumacaftor/ivacaftor reduced sweat chloride concentration by 32 mmol/L in *F508del* homozygous children aged 2–5 years and only 18 mmol/L in adolescents and adults (McNamara et al., 2019; Stahl et al., 2024b; Graeber et al., 2018). In addition, lumacaftor/ivacaftor restored CFTR function in the rectal epithelium to approximately 30% of normal CFTR activity in *F508del* homozygous children aged 2–11 years (Berges et al., 2023), whereas in adolescents and adults, functional improvement was more modest, in the range of 10%–20% (Graeber et al., 2018). These findings suggest that younger people with CF may have a greater potential for CFTR rescue, however, the mechanisms underlying this age-dependent response are currently unknown.

Intestinal current measurement (ICM) was developed as a sensitive technique to assess CFTR-mediated chloride transport in the intestinal epithelium *ex vivo* (De Jonge et al., 2004; Hirtz et al., 2004; Sousa et al., 2012; Veeze et al., 1991; Mall et al., 1998b; Veeze et al., 1994; Mall et al., 2000a). By measuring the change in transepithelial short-circuit current in response to cyclic adenosine monophosphate (cAMP)-mediated as well as cholinergic stimulation, ICM provides a direct readout of CFTR function in the intestinal epithelium (Figure 1). Early studies using Ussing chamber experiments on rectal tissues were pioneering in the field of CF research providing valuable insights into the pathophysiology of CFTR dysfunction (Mall et al., 1999; Veeze et al., 1991; Mall et al., 1998b; Veeze et al., 1994; Mall et al., 2000a; Mall et al., 2002; Mall et al., 2004a; Mall et al., 2000b; Roth et al., 2011). Further, ICM was established as a diagnostic tool and is used to determine the effects of CFTR modulator therapies on CFTR function (Graeber et al., 2018; De Jonge et al., 2004; Hirtz et al., 2004; Sousa et al., 2012; Clancy et al., 2013; Graeber et al., 2015). Despite significant advances in understanding CFTR function across different epithelial tissues, there is still limited knowledge about the age dependency of CFTR function.

The primary objective of this study was, therefore, to investigate whether CFTR function in the intestinal epithelium exhibits age-dependent variability. To achieve this, we conducted a comprehensive analysis of CFTR-mediated chloride secretion in rectal biopsies from 258 people without CF and 72 people with CF, ranging in age from 1 month to 68 years. Additionally, we performed quantitative real-time PCR to assess the expression of *CFTR* and conducted morphometric analyses of the crypts in the intestinal epithelium to determine whether structural changes in the epithelium could explain differences in CFTR function across age groups. To test the hypothesis that the response to CFTR modulator therapy is age-dependent, we performed a secondary analysis of our previous studies on the effects of lumacaftor/ivacaftor on CFTR function in different age groups (Graeber et al., 2018; Berges et al., 2023).

## 2 Methods

### 2.1 Study participants

This retrospective study was approved by the Ethical Committees at the University Hospitals of Heidelberg and Freiburg and the Charité - Universitätsmedizin Berlin. Written informed consent was obtained from all participants included in the study, their parents or legal guardians. ICM was performed in 258 people without CF and 72 people with pancreatic-insufficient CF between 1997 and 2022. The diagnosis of CF was established by clinical symptoms characteristic of CF, increased sweat chloride concentrations (≥60 mmol/L) and/or detection of two disease-causing *CFTR* mutations. People with CF did not receive any CFTR modulator therapy at the time of the rectal biopsy. People without CF had a sweat chloride concentration below 60 mmol/L and the diagnosis of CF was excluded by a CF physician. People without CF and people with CF were grouped in different age groups according to the American Academy of Pediatrics (Hagan et al., 2017). The correlation of the response to CFTR modulator therapy with lumacaftor/ivacaftor and age was performed as a secondary analysis of our previous studies on the effects of lumacaftor/ivacaftor on CFTR function in 49 F508del homozygous people with CF aged 12 years and older (Graeber et al., 2018) and 12 children aged 2–11 years (Berges et al., 2023).

### 2.2 Intestinal current measurements

ICM was performed as previously described (Graeber et al., 2022a; Graeber et al., 2018; Hirtz et al., 2004; Graeber et al., 2015; Mall et al., 2004b). In brief, superficial biopsies of the rectal mucosa (∼2–3 mm in diameter) were collected by endoscopic forceps biopsy and immediately stored in ice cold tissue medium (medium 199 containing Hank’s salts, L-glutamine and 25 mmol/L HEPES complemented with 5 mmol/L glycine and 0.5 mmol/L Sodium-DL-β-hydroxybutyrate). Rectal biopsy specimens were mounted in perfused micro-Ussing chambers (open area ∼0.95 mm^2^). The luminal and basolateral surfaces of the epithelium were perfused continuously with a bath solution of the following composition (mmol/L): 145 NaCl, 0.4 KH2PO4, 1.6 K2HPO4, 5 D-glucose, 1 MgCl2, and 1.3 calcium gluconate, pH 7.4, at 37°C. Experiments were performed under open-circuit conditions. Values for the transepithelial voltage (V_te_) were referenced to the serosal surface of the epithelium. Transepithelial resistance (R_te_) was determined by applying intermittent (1 s) current pulses (ΔI = 0.5 µA). The equivalent short-circuit current (I_sc_) was calculated according to Ohm’s law from V_te_ and R_te_ (I_sc_ = V_te_/R_te_) after appropriate correction for fluid resistance. The resistance of the rectal epithelium did not change with age (r = 0.000, P = 0.815; Supplementary Figure S1).

Rectal tissues were equilibrated for 40 min in the presence of amiloride (10 μmol/L, luminal) to block electrogenic sodium absorption and indomethacin (10 μmol/L, basolateral) to inhibit prostaglandin E2 synthesis and endogenous cAMP formation. 3-Isobutyl-1-methylxanthine (IBMX) and forskolin (100 μmol/L and 1 μmol/L, basolateral) were added to obtain maximal cAMP-mediated activation of CFTR as previously described (Figure 1) (Graeber et al., 2022a; Graeber et al., 2018; Hirtz et al., 2004; Graeber et al., 2015; Mall et al., 2004b). To increase the driving force for chloride secretion by CFTR, we determined the responses to carbachol (100 μmol/L, basolateral) after stimulation with IBMX/forskolin. The concentrations used for forskolin and carbachol were based on previous studies assessing a dose-response curve to result in maximal activation of I_sc_ (Strohmeier et al., 1995; McNamara et al., 1999; Kerr et al., 1995). To control for sample-to-sample variability, bioelectric measurements were performed on 2–5 biopsy specimens per individual, and data were averaged to obtain a single value for each individual. Indomethacin, amiloride, IBMX, forskolin, and carbachol were all obtained from Sigma-Aldrich (Taufkirchen, Germany).

### 2.3 Real-time PCR

Rectal biopsies were stored in RNAlater (Invitrogen, Darmstadt, Germany), total RNA was isolated using the RNeasy Mini Kit (Qiagen, Hilden, Germany) and reverse transcribed into cDNA using Superscript III (Invitrogen, Darmstadt, Germany). Quantitative RT-PCR for *CFTR* and *GAPDH* was performed on an Applied Biosystems 7,500 Real Time PCR System using TaqMan universal PCR master mix and inventoried TaqMan gene expression assays according to the manufacturer’s instructions (Applied Biosystems, Darmstadt, Germany). Relative fold changes in target gene expression were calculated from the efficiency of the PCR reaction and the crossing point deviation between samples from the two age groups, and determined by normalization to expression of the reference gene *GAPDH*, as previously described (Mall et al., 2008; Zhou et al., 2008).

### 2.4 Morphometric analysis

Rectal tissues were embedded in O.C.T. (Sakura Finetek Europe, Umkirch, Germany) and stored at −80°C until further processing. Thin sections (6–8 µm) of frozen rectal tissues were cut and mounted on glass slides. Sections were fixed in 10% buffered formalin for 30 min at room temperature and subsequently stained with hematoxylin and eosin. The length of nine crypts from at least three different sections of the biopsies was measured. Only crypts with a luminal opening and reaching to the serosa were selected for measurements. The total number of cells was determined by counting the number of hematoxylin positive nuclei. Goblet cells were defined by absence of staining and non-goblet cells were calculated by subtracting the number of goblet cells from the number of total cells.

### 2.5 Statistical analysis

Data were analyzed using GraphPad Prism 9.5.1 (GraphPad Software, San Diego, CA, United States of America) and SigmaPlot 12.5 (Grafiti LLC Palo Alto, CA, United States of America). Data are presented as mean and standard error of the mean (SEM) and were tested by Student’s t-test, Mann-Whitney Rank Sum test or one-way ANOVA with Dunn’s *post hoc* test as appropriate. Correlations were assessed using and Spearman correlation coefficient. P < 0.05 was accepted to indicate statistical significance.

## 3 Results

### 3.1 CFTR-dependent chloride secretion in native rectal epithelia decreases with age

**FIGURE 2:**
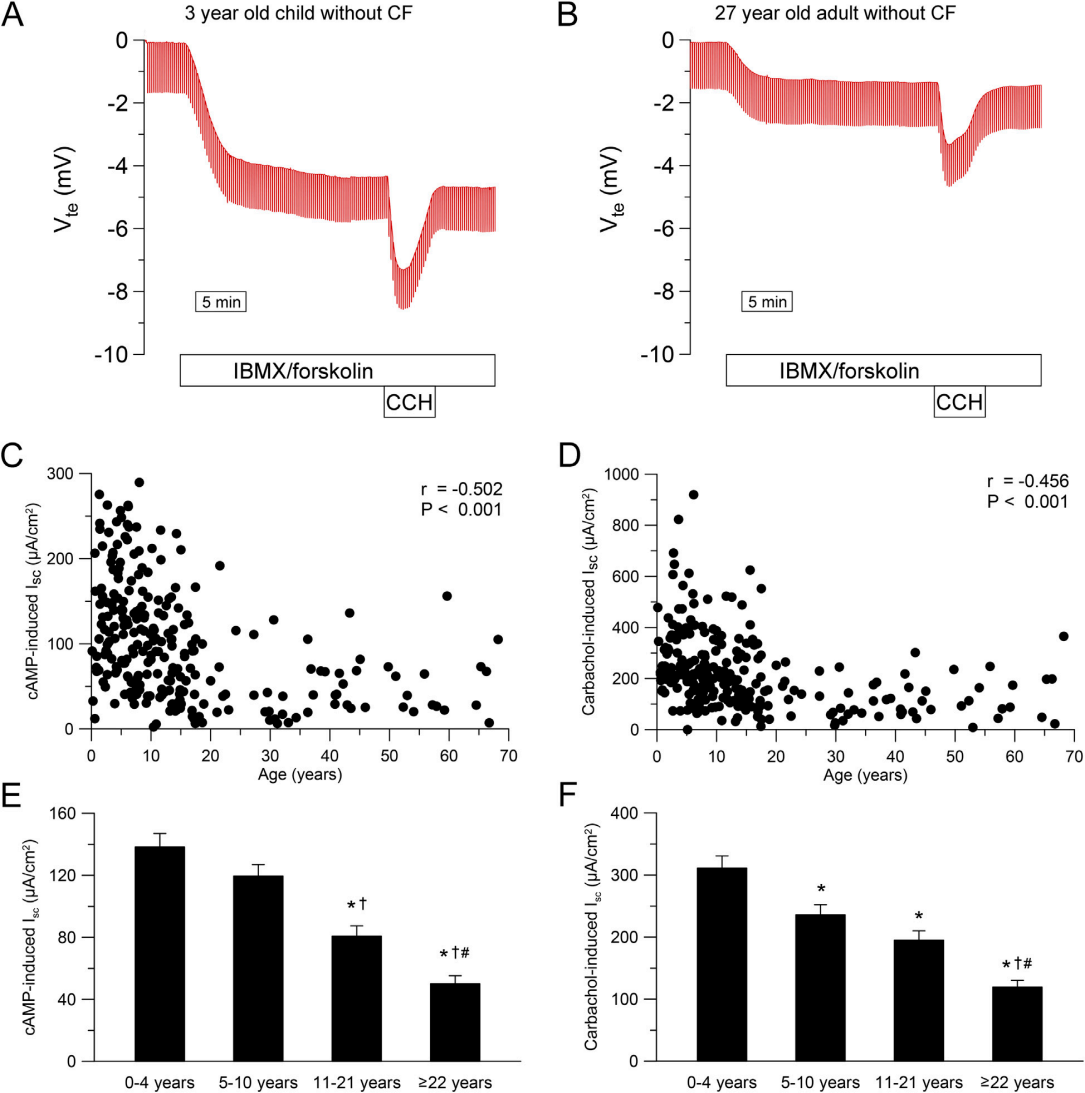
Chloride secretion in human rectal tissue is age-dependent. **(A, B)** Original recordings of the effects of cAMP-dependent (100 μmol/L IBMX and 1 μmol/L forskolin, basolateral) and cholinergic (100 μmol/L carbachol, basolateral) activation on V_te_ and R_te_ in rectal tissue from **(A)** A 3 year old and **(B)** A 27 year old person without CF. **(C, D)** Summary of the effects of **(C)** cAMP-induced (IBMX/forskolin) and **(D)** Carbachol-induced short-circuit current (I_sc_) in rectal tissues from people without CF. **(E, F)** Summary of the effects of **(E)** cAMP-induced and **(F)** carbachol-induced short-circuit currents in rectal tissues from people without CF in different age groups. Experiments were performed in the presence of indomethacin and amiloride. n = 258; *, P < 0.05 vs. 0–4 years, †, P < 0.05 vs. 5–10 years, #, P < 0.05 vs. 11–21 years.

**FIGURE 3:**
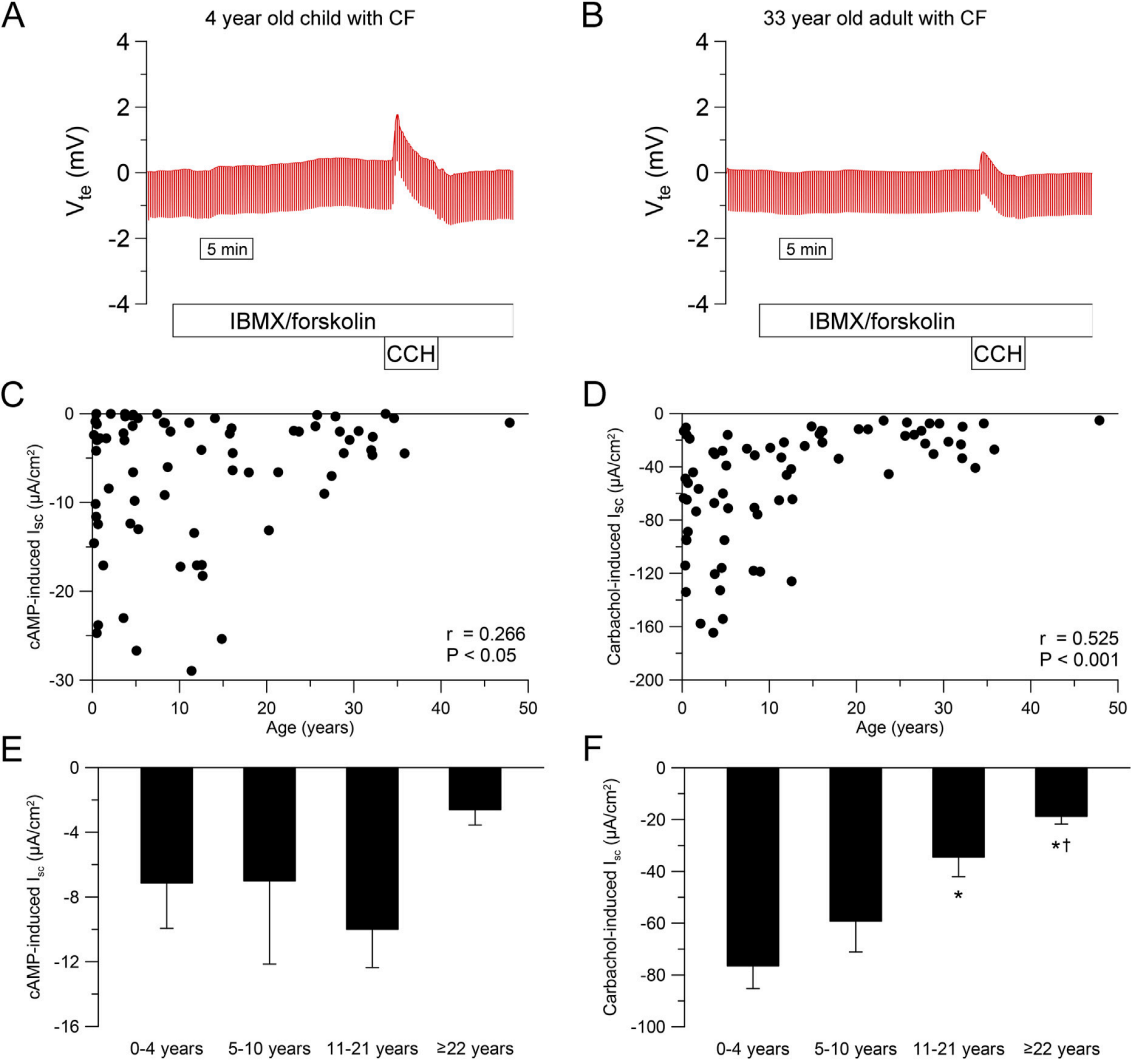
Ion transport in cystic fibrosis rectal tissue in different age groups. **(A, B)** Original recordings of the effects of cAMP-dependent (100 μmol/L IBMX and 1 μmol/L forskolin, basolateral) and cholinergic (100 μmol/L carbachol, basolateral) activation on V_te_ and R_te_ in rectal tissue from **(A)** A 4 year old and **(B)** a 33 year old person with CF. **(C, D)** Summary of the effects of **(C)** cAMP-induced (IBMX/forskolin) and **(D)** Carbachol-induced short-circuit current (I_sc_) in rectal tissues from people with CF. **(E, F)** Summary of the effects of **(E)** cAMP-induced and **(F)** carbachol-induced short-circuit currents in rectal tissues from people with CF in different age groups. Experiments were performed in the presence of indomethacin and amiloride. n = 72; *, P < 0.05 vs. 0–4 years, †, P < 0.05 vs. 5–10 years.

To study the age-dependency of CFTR-dependent chloride secretion in native human rectal epithelia, we performed ICM in 258 people without CF and 72 people with CF with an age ranging from 1 month to 68 years. In infants and preschool children, we observed a greater response to IBMX/forskolin (cAMP-induced short-circuit current (I_sc_)) and carbachol compared to adults without CF (Figures 2A,B). This age dependency in people without CF was especially observed during childhood and adolescence with decrease over time for cAMP-induced response (r = −0.502, P < 0.001, Figure 2C) and carbachol-induced response (r = −0.456, P < 0.001, Figure 2D). By categorizing people without CF in age groups, we observed a reduction in cAMP- and carbachol-induced responses across age ranges (Figures 2E,F). cAMP-induced responses in infants and preschool children (0–4 years) and school children (5–10 years) without CF were higher compared to adolescents (11–21 years) and adults (≥22 years) (each P < 0.05, Figure 2E). In addition, cAMP-induced responses in adults was smaller compared to adolescents without CF (P < 0.05, Figure 2E). Similarly, Carbachol-induced responses in infants and preschool children (0–4 years) without CF were higher compared to school-age children (5–10 years), adolescents (11–21 years) and adults (≥22 years) (each P < 0.05, Figure 2F). Furthermore, carbachol-induced responses in adults was smaller compared to adolescents as well as school -age children without CF (both P < 0.05, Figure 2F). In people with CF, cAMP- and carbachol-induced negative I_sc_ responses reflect potassium secretion (Figures 3A,B) (Kunzelmann and Mall, 2002; Mall et al., 2000a; Mall et al., 2004b). We observed a weak correlation between cAMP- induced I_sc_ and age (r = 0.266, P < 0.05; Figure 3C), but cAMP-induced responses were overall small and did not differ across age groups (Figure 3E). Carbachol-induced potassium secretory responses decreased with age in people with CF (r = 0.525, P < 0.01; Figure 3D). Adolescents (11–21 years) with CF had lower carbachol-induced responses compared to infants and preschool children (0–4 years) and adults (≥22 years) exhibited lower carbachol-induced responses compared to infants and preschool (0–4 years) as well as school-age children (5–10 years) (all P < 0.05, Figure 3F).

### 3.2 CFTR mRNA increases with age

Next, we determined the effect of aging on mRNA transcript levels of CFTR in rectal tissues by quantitative real-time PCR. The expression level of *CFTR* mRNA was higher in adults (≥22 years) compared to infants and preschool children without CF (≤4 years) (P < 0.05; Supplementary Figure S2).

### 3.3 Non-goblet cells in the lower half of the crypt are reduced with age

**FIGURE 4:**
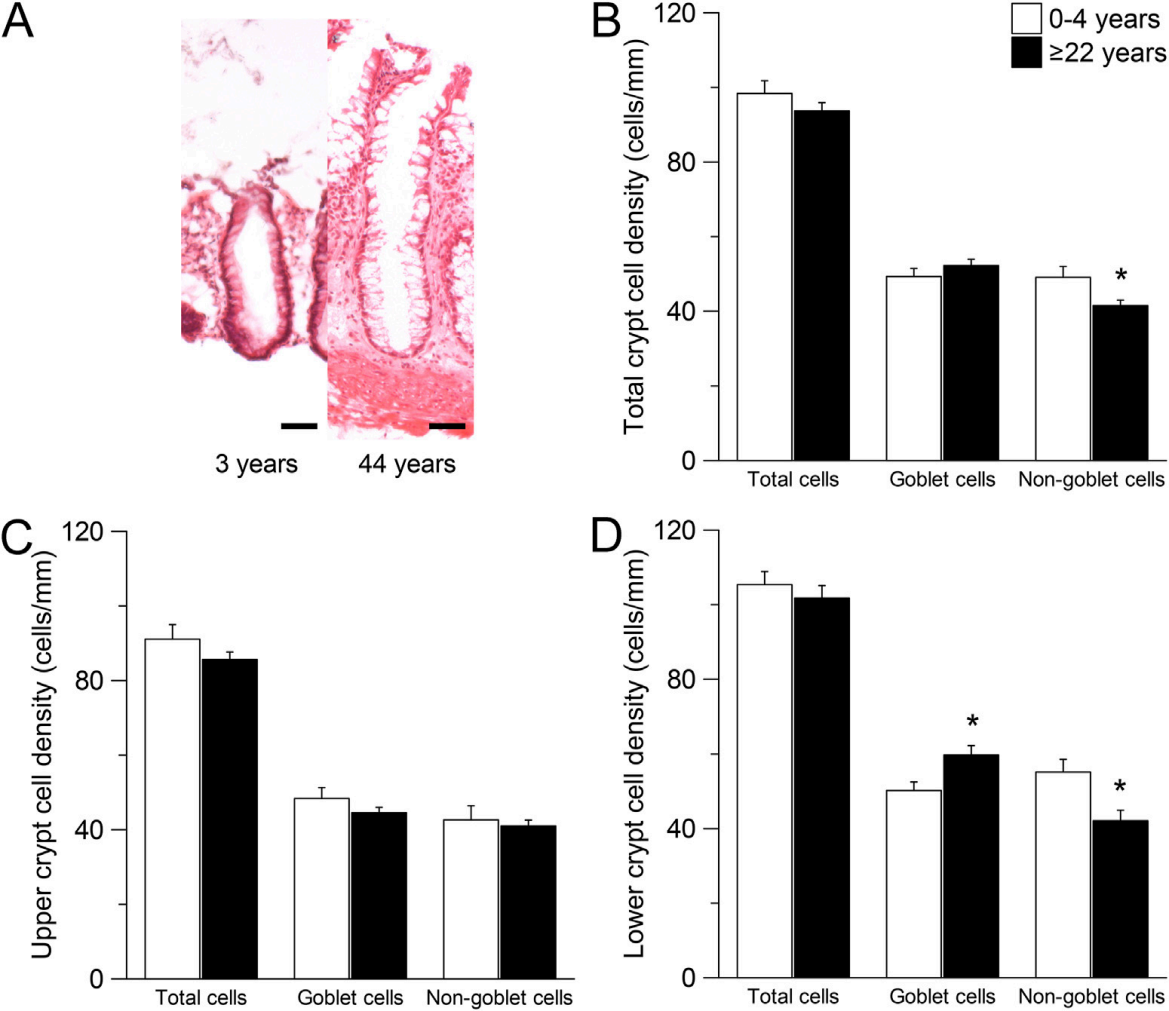
Morphometric analyses of the intestinal crypts in children compared to adults. **(A)** Morphology of crypts in rectal biopsies of people without CF with the age of 3 years and 44 years. Sections were stained with hematoxylin and eosin (H&E). Scale bars = 50 µm. **(B–D)** Total cell, goblet cell and non-goblet cell counts from **(B)** The total crypt **(C)** the upper crypt half and **(D)** The lower crypt half of people without CF aged 0–4 years and ≥22 years. n = 9; *, P = 0.05.

To investigate age-dependent differences in epithelia cell type composition of the rectal epithelium, we examined the crypt morphology in H&E stained sections of rectal biopsies from infants and preschool children (≤4 years) and adults (≥22 years) without CF (Figure 4A). There was a lower number of non-goblet cells in the whole crypt of adults compared to infants and preschool children (P < 0.05), but no difference was observed in the number of total cells and goblet cells (Figure 4B). Since CFTR was shown to be mostly expressed in non-goblet cells at the crypt base (Greger et al., 1997; Greger, 2000; Kunzelmann and Mall, 2002; Ecke et al., 1996; Linley et al., 2014), we performed a regional sub analysis investigating the upper and the lower half of the crypt. There was no difference in the number of any cell type in the upper half of the crypt (Figure 4C). However, in the lower half of the crypt, the number of goblet cells was increased and the number of non-goblet cells was decreased in adult compared to infants and preschool children without CF (both P < 0.05), whereas no change in the total number of cells was observed (Figure 4D).

### 3.4 Response to CFTR modulator therapy lumacaftor/ivacaftor decreases with age

**FIGURE 5:**
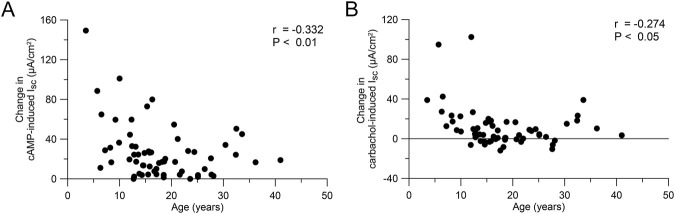
Response to CFTR modulator therapy is age-dependent. **(A, B)** Change in cAMP-induced **(A)** and carbachol-induced **(B)** short circuit current in rectal tissue of people with CF on lumacaftor-ivacaftor therapy compared to baseline (n = 61). Data was reanalyzed from Graeber et al. (2018); Berges et al. (2023).

To test the hypothesis that the response to CFTR modulator therapy is age-dependent, we performed a secondary analysis of the ICM data of our previous studies on the effects of lumacaftor/ivacaftor on CFTR function in F508del homozygous people with CF aged 12 years and older (Graeber et al., 2018) and 2–11 year old children (Berges et al., 2023). The change in cAMP-induced I_sc_ after initiation of lumacaftor/ivacaftor compared to baseline correlated with the age of people with CF (r = −0.333, P < 0.01; Figure 5A). Similarly, the change in carbachol-induced I_sc_ after initiation of lumacaftor/ivacaftor decreased with age (r = −0.277, P < 0.05; Figure 5B)

## 4 Discussion

### 4.1 CFTR function declines with age in the rectal epithelium

To our knowledge, this is the first study assessing CFTR function in the rectal epithelium across different age groups. Our data show that CFTR-dependent chloride transport in people without CF decreases with age, particularly in childhood and adolescence (Figure 2). Additionally, we observed reduced potassium secretion with increasing age in people with CF (Figure 3). As a decrease in potassium secretion increases the net current, the age dependent decline in chloride transport could be slightly underestimated. Interestingly, we observed an increase in *CFTR* mRNA levels in older compared to younger people without CF (Supplementary Figure S2). We hypothesize that the increase in *CFTR* mRNA is caused by a feedback mechanism trying to compensate for the functional decline. However, the sensitivity of the whole tissue PCR is unclear as CFTR levels in the colon are lower compared to other parts of the intestine (Busslinger et al., 2021; Burclaff et al., 2022; Elmentaite et al., 2021). This further suggests that the observed functional decline is not due to reduced transcription with age but may result from tissue remodeling over time. Enterocytes in the crypt base have been described as the major contributors to cAMP-mediated chloride secretion in the colon (Greger et al., 1997; Greger, 2000; Kunzelmann and Mall, 2002; Ecke et al., 1996; Linley et al., 2014). We demonstrate that morphological changes with a reduced number of non-goblet cells especially in the lower crypt are present in older people without CF (Figure 4). These changes may reflect age-related epithelial remodeling, which has been implicated in other studies studying the colon (Tran and Greenwood-Van Meerveld, 2013) and may explain the age-dependent decrease in CFTR-dependent chloride secretion. In addition to morphological changes in the crypt epithelium, the age-dependent decrease in CFTR-dependent chloride secretion may also be associated with age-related alterations in the efficiency of CFTR biogenesis, including folding and trafficking or degradation of CFTR proteins, as well as changes in the cellular CFTR regulation. To the best of our knowledge, there is no clinical evidence regarding altered intestinal secretory function under healthy conditions with increasing age. This lack of evidence may be attributed to compensation mechanisms, such as reduced efficiency of water and electrolyte absorption. Nonetheless, the reduced maximal capacity for cAMP-dependent chloride secretion in older individuals may also remain sufficient to maintain normal intestinal function under physiological conditions but may lower the threshold for the development of pathological conditions, as discussed below.

### 4.2 Age-dependent response to CFTR modulator therapy

Clinical trials as well as real world observational studies consistently demonstrated larger effects of CFTR modulator therapy on CFTR function in children compared to adolescents and adults with CF (Middleton et al., 2019; Heijerman et al., 2019; Goralski et al., 2023; McNamara et al., 2019; Stahl et al., 2024b; Graeber et al., 2018; Berges et al., 2023; Boyle et al., 2014; Stahl et al., 2023; Nichols et al., 2022; Mall et al., 2022). Therapy with ETI and lumacaftor/ivacaftor leads to more pronounced sweat chloride reductions in children compared to adults with CF (Middleton et al., 2019; Heijerman et al., 2019; Goralski et al., 2023; McNamara et al., 2019; Stahl et al., 2024b; Graeber et al., 2018). Furthermore, our secondary analysis of previous studies assessing the effects of lumacaftor/ivacaftor in different age groups of F508del homozygous people with CF (Graeber et al., 2018; Berges et al., 2023) supports an age dependent decrease of functional restoration (Figure 5). Of note, pharmacokinetic profiles of CFTR modulators in children were generally consistent with those observed in older patients (Goralski et al., 2023; McNamara et al., 2019; Zemanick et al., 2021). Therefore, our observation of higher baseline CFTR function in younger people may provide a mechanistic basis for the age-dependent response to CFTR modulators and suggests a greater potential of functional restoration in younger age groups (Goralski et al., 2023; McNamara et al., 2019; Stahl et al., 2024b; Berges et al., 2023; Stahl et al., 2023; Mall et al., 2022). Interestingly, a recent study of bulk and single-cell sequencing data from lung epithelium also suggests an age-dependent decline in CFTR function in the lungs (Corcoran et al., 2024). Our findings support the importance of initiating CFTR modulator therapy early in life to maximize long-term therapeutic efficacy, as younger patients may benefit from rescue of higher levels of CFTR function and less structural epithelial remodeling and organ damage, two factors that may facilitate restoration of epithelial homeostasis, as recently supported by single cell RNA sequencing studies of nasal epithelial cells from children with CF who initiated ETI therapy (Loske et al., 2024).

### 4.3 Potential role of age-dependent CFTR function in secretory diarrhea and chronic constipation

Our findings may also have implications for understanding the role of CFTR in secretory diarrhea, the third leading cause of death in children under 5 years (WHO, 2024; Hartman et al., 2023). CFTR-mediated chloride and water secretion are critical in maintaining intestinal fluid homeostasis (Mall et al., 1998a; Greger et al., 1997; Greger, 2000; Kunzelmann and Mall, 2002; Abraham and Taylor, 2017; Geibel, 2005). Enterotoxins activate CFTR channels to drive excessive chloride-driven fluid secretion, leading to severe dehydration and electrolyte imbalances (Thiagarajah et al., 2015). The higher CFTR function in infants and pre-school children may aggravate these pathologies contributing to an increased volume loss and morbidity and mortality in this age group. Conversely, older people with diminished CFTR function may experience less severe fluid loss and symptoms during acute intestinal infections, but may conversely be more prone to develop constipation. While chronic constipation has a complex, multifactorial etiology, CFTR plays a pivotal role as a regulator of intestinal ion and fluid balance and serves as a therapeutic target in constipation management (Greger et al., 1997; Greger, 2000; Kunzelmann and Mall, 2002; Black and Ford, 2018). In people with CF, reduced chloride secretion results in an increased susceptibility of constipation and severe complications such as DIOS (Kunzelmann and Mall, 2002; Abraham and Taylor, 2017). The age-related decline in CFTR function observed in our study may therefore contribute to the higher prevalence of constipation observed in older people (Choung et al., 2007).

### 4.4 CFTR function and colorectal cancer

Furthermore, CFTR dysfunction in the intestinal epithelium has been linked to a higher susceptibility for CRC (Spelier et al., 2024), the third most common cancer with high mortality (Bray et al., 2024). Epidemiological studies suggest that people with CF have a 5 times greater risk of developing CRC compared to the general population (Birch et al., 2023). Interestingly, also people who are only carriers of CFTR mutations have a higher probability of developing CRC suggesting that even minimal CFTR dysfunction may contribute to the complex multifactorial pathophysiology of CRC (Shi et al., 2021). The elevated risk of CRC in people with CF is not yet completely understood but CFTR dysfunction in the intestine is known to be associated dysbiosis of the gut microbiome and chronic inflammation, two factors that have been associated with the development of intestinal cancer (Li et al., 2024; Munn, 2017). Furthermore, several studies suggest that *CFTR* itself functions as a tumor suppression gene (Than et al., 2017; Amaral et al., 2020; Liu et al., 2020; Scott et al., 2023). Our findings of an age-dependent decline in CFTR function in people without CF may therefore contribute to an increased risk of developing CRC in older people as the majority of CRC patients are diagnosed after the age of 65 years (Siegel et al., 2023).

### 4.5 Limitations

This study also has some limitations: The cross-sectional design of our study limits the ability to track longitudinal changes in CFTR function and epithelial morphology in individuals, which would provide more detailed insights into the progression of age-related changes. Further, we assessed CFTR function in rectal tissue only and it is unknown if the observed changes with age are tissue-specific and how CFTR function decreases with age in other organs in which CFTR plays important roles in health and disease, especially the lungs. Finally, potential confounding factors such as diet, comorbidities, and prior treatment history were not explicitly controlled for in this study, which may influence the observed age-dependent changes in CFTR function and crypt morphology. Future studies addressing these limitations, including longitudinal analyses and investigations in other tissues will be important to confirm these findings.

### 4.6 Conclusion

This study is the first to demonstrate an age-dependent decline in CFTR-mediated chloride transport in the intestinal epithelium, identifying morphological changes in the crypt epithelium as a potential mechanism. These results provide a mechanistic basis for age-dependent differences in CFTR modulator efficacy and offer new perspectives on the pathophysiology of diseases such as secretory diarrhea and chronic constipation. Our findings suggest that early initiation of CFTR modulator therapies may yield the greatest therapeutic potential for people with CF.

## Data Availability

The datasets presented in this article are not readily available because Publication or accessibility of patient-related data beyond what is represented above is not permitted due to local data protection regulations and ethics guidelines. Requests to access the datasets should be directed to simon.graeber@charite.de.
