# Supplementary material for: Intestinal current measurement detects age-dependent differences in CFTR function in rectal epithelium
Source: Front Pharmacol. 2025 Feb 24;16:1537095. doi: 10.3389/fphar.2025.1537095 (PMC11891205; doi:10.3389/fphar.2025.1537095)
Supplement: Supplementary file 1 [file DataSheet1.docx]

Supplementary Material

# Supplementary Figures


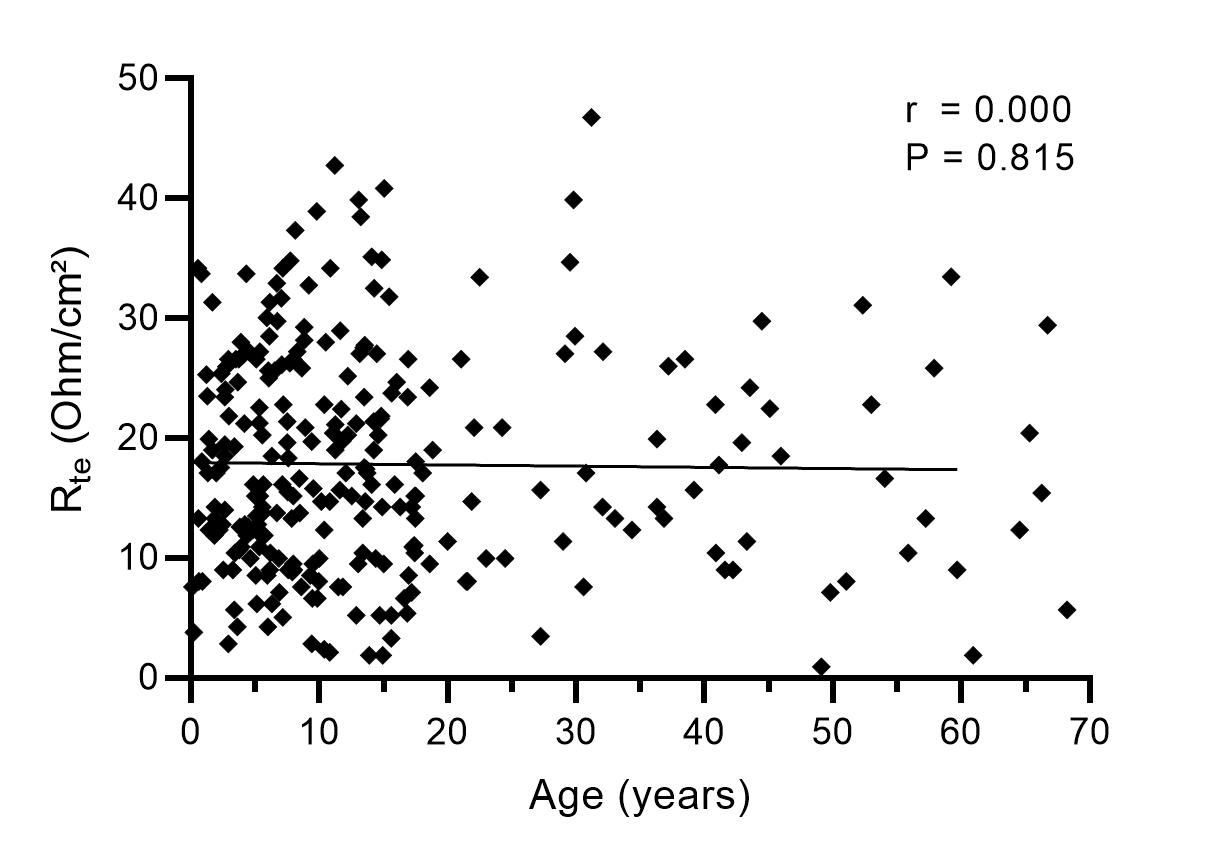


**Supplementary Figure S1.** Transepithelial resistance (R_te_) of human rectal tissue from people without CF, n = 258.

**
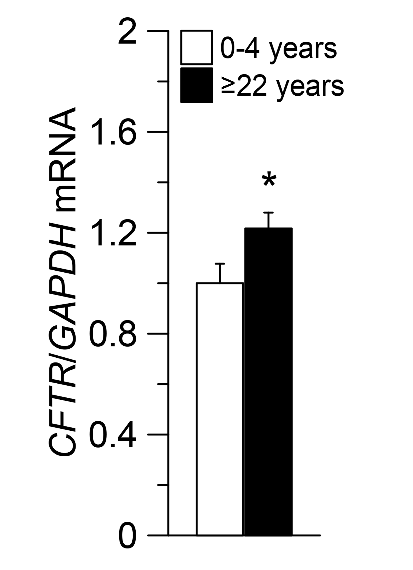
**

**Supplementary Figure S2.** **Expression of CFTR in rectal tissues from children compared to adults.** Transcript levels of CFTR in rectal tissues from people without CF aged 0 - 4 years and ≥ 22 years. Data are expressed as fold changes over people aged 0 - 4 years. n = 8 per group. *, P< 0.05.
